# Editorial: Children and Adolescent Quality of Life under Socialism with Chinese Characteristics

**DOI:** 10.1007/s11482-021-09999-3

**Published:** 2021-09-22

**Authors:** Daniel T. L. Shek, Huamin Peng, Zheng Zhou

**Affiliations:** 1 Department of Applied Social Sciences, The Hong Kong Polytechnic University, Kowloon, Hong Kong; 2 Department of Social Work and Social Policy, Nanjing University, Nanjing, China; 3 Research Institute of Social Development, Southwestern University of Finance and Economics, Chengdu, China

Since most of the social science studies are conducted in Western contexts, there is a criticism that theories and findings based on such studies are biased in nature. With particular reference to psychological studies, Henrich et al. (2010) pointed out that psychological knowledge is based on “WEIRD” samples, with participants commonly recruited from “Western, educated, industrial, rich and democratic” societies. This criticism is also applicable to research studies on quality of life (QOL) where “WERID” samples are commonly used to understand quality of life in different populations. Conceptually speaking, findings based on “WEIRD” samples may not be generalizable because they are not representative of the “whole” human population and the behavior under examination is constrained by the unique social, economic, political and cultural contexts of the studies. For example, while Western theorists commonly assert that parental psychological control impairs child development, the relationship between psychological control and child developmental outcomes does not work in a linear manner (Leung & Shek, 2020). Methodologically speaking, Western measures of quality of life may not be wholly applicable to non-Western contexts. In response to the predominance of research studies based on “WEIRD” samples, there is a need to conduct research based on “non-WEIRD” populations, particularly Chinese people.

There are several arguments why more Chinese quality of life studies are needed. Primarily, the huge population size of Chinese people strongly justifies the need for Chinese research. In the latest census in China, the population was more than 1.4 billion (National Bureau of Statistics of China, 2021), which is roughly 18% of the global population (World Population Prospects, 2019). Such a huge number implies that the generalizability of QOL theories and research findings must make reference to Chinese people. As pointed out by Rad et al. (2018), journal editors “should instruct reviewers to treat non-WEIRDness as a marker of the interest and importance of a paper” (p. 11404).

Besides, with reference to the attributes of “WEIRD” studies, the Chinese population is “non-WEIRD.” First, Chinese culture is a non-Western culture. Second, higher education is still not widespread. Third, the degree of industrialization in China is moderate, which accounts for 37.8% of the economic value of China in 2020 (Statista, 2021). Fourth, China cannot be regarded as a “rich” country. The GDP per capita of China in 2020 was roughly US$10,500.4, which was below the average world GDP per capita (US$10,925.7; The World Bank, 2020). According to the Communist Party of China, China can only be regarded as a “xiao kang” society (i.e., a moderately prosperous society in all aspects). Finally, the Chinese Government claimed that the Chinese political system is a “Chinese style” democracy, although its operation is unique when compared to contemporary Western democratic systems (Lee, 2010).

There are three aspects of China deserving our attention. The first aspect is the cultural roots of China which have shaped the socialization process. In the traditional Chinese culture, dominant social ideologies were Confucian, Buddhist and Taoist philosophies, which emphasized both inner and interpersonal harmony, self-cultivation, and moral order. Besides, while the collective interest occupies a more important role over the individual interest in Confucian thoughts, acceptance of fate and “natural order” is central to Buddhism and Taoism. Based on this cultural heritage, Chinese parents typically possess several characteristics, including parent-centeredness, use of harsh socialization practices, and emphasis on filial piety in children (Shek & Sun, 2014).

Besides, there are some “desired” attributes of children and adolescents in the traditional Chinese culture, particularly under Confucianism. In the “Standards for Being a Good Student and Child” (*Di Zi Gui*), there are developmental tasks of “optimal” development of children, such as trustworthiness and virtues. In an attempt to examine *Di Zi Gui* with reference to contemporary parenting science, Shek and Law (2019) concluded that some developmental tasks outlined in *Di Zi Gui* are in fact consistent with the assertions in the contemporary parenting literature (e.g., importance of bonding and character development), although there is divergence in the related views on children’s rights. They concluded that “although *Di Zi Gui* is a classic Chinese text written several centuries ago, some assertions show striking resemblance to the contemporary scientific findings and it has tremendous implications for contemporary youth development and parenting science and practice” (Shek & Law, 2019, p. 403). In another study exploring Chinese ideals of child and adolescent development, Shek et al. (2013) outlined the related attributes in Confucian thoughts, including “zhong” (loyalty), “xiao” (filial piety), “ren” (benevolence), “ai” (love), “xin” (trustworthiness), “yi” (righteousness), “he” (harmony), “li” (propriety), “zhi” (wisdom), “lian” (incorrupt), and “chi” (shame). They also pointed out that Confucian virtues are similar to some of the character strengths emphasized in contemporary positive psychology literature. In contemporary Chinese societies, children are socialized to possess academic excellence, proper behavior and obedience (Shek & Sun, 2014). In particular, morbid emphasis on academic excellence is a hallmark of the development of Chinese children and adolescents (Shek & Siu, 2019).

The second unique aspect of China is its political system – Socialism with Chinese characteristics. On the one hand, there are views “demonizing” the influence of the Communist political system on the “healthy” development of Chinese children and adolescents. The argument is that the Socialist political system over-emphasizes the collective interest of the State over individual rights. There is also the criticism that over-control of the State constitutes a poor developmental context for optimal child development, such as undermining their autonomy and creativity. On the other hand, there are views suggesting that harmony and stability brought forth by the Communist system is indeed beneficial to the development of well-being in children and adolescents. In fact, in the past four decades, more than 700 million people have escaped from the poverty trap, which has substantially reduced the detrimental impact of economic disadvantage on children and adolescent quality of life. There is also a recent emphasis on “shared prosperity” (not “shared poverty”) in Socialist China. Furthermore, there are views showing that the Chinese Communist system is effective in fostering social development (i.e., no need to waste time on “unnecessary” political discussion). This argument is clearly supported under the COVID-19 where China is the first country being successfully recovered from the pandemic (Shek, 2021). Finally, there is another argument that if the Communist system is so bad, then it would be puzzling to learn that Chinese people travelling outside China have rarely sought political asylum in recent years. In fact, many Chinese tourists go back to China voluntarily after travelling outside China.

Finally, it should be noted that there have been drastic economic and social changes in China as a result of the Reform and Opening-up Policy since the late 1970s. Economically speaking, China has gradually transited from a totally centrally planned economy to an economy which has the features of the free economy and State control. Because of the economic growth, quality of life of Chinese people has substantially improved in the past few decades. At the same time, the rapid economic growth has also widened the rich-poor divide. Because of the economic growth in the large cities, “left-behind” children in rural areas has become a social issue. Socially speaking, the social structure of China has changed by the emergence of the middle class, an increase in people with higher educational attainment, and a decline in illiteracy rate. Paradoxically, such social changes have contributed to the rise in divorce, single parenthood and re-marriage rates which have created quality of life issues for children, adolescents and the family.

With the above backdrop, the field of quality of life definitely needs more Chinese QOL research. Although there are more recent research studies on Chinese QOL in children and adolescents, particularly those published in *Applied Research in Quality of Life* (e.g., Li et al., 2019; Lu et al., 2021; Zhou et al., 2020), such research is unsystematic. As such, we published a special issue entitled “Quality of life among children and adolescents in Chinese Societies” with most of the papers were based on adolescents in Hong Kong (Leung & Fung, 2021). While the papers in this special issue are valuable and there are similarities between Hong Kong and mainland China (e.g., strong emphasis on academic excellence), it may not allow QOL researchers to fully understand the well-being of children and adolescents in mainland China because there are differences between Hong Kong and mainland China in several areas. Economically speaking, while Hong Kong is basically a service economy, mainland China’s economy is contributed by agricultural, industrial and service activities. Besides, while State involvement is strong in mainland China, Hong Kong upholds the philosophy of laissez-faire. Politically speaking, while Communism guides the development in China, Hong Kong’s political system is a hybrid structure governed by the Basic Law and Western election mechanisms. Socially speaking, while it is more community oriented in mainland China, individual orientation is more emphasized in Hong Kong. Culturally speaking, while mainland Chinese culture is shaped by Chinese and Socialist ideologies, Hong Kong culture is influenced by Chinese and Western ideologies, particularly Capitalist ideologies. Finally, based on the Human Development Index Report (2020), Hong Kong’s rank (4/189) is higher than China’s rank (85/189). This suggests a need to look at the quality of life of children and adolescents in depth. Unfortunately, research on QOL in children and adolescents is isolated and unsystematic. Therefore, we have decided to have a special issue entitled “Quality of life of children and adolescents under Socialism with Chinese characteristic” guest edited by Huamin Peng and Zheng Zhou. As Professor Peng was seriously ill and unable to spend much time writing this editorial, I stepped in to help complete the editorial of this special issue.

There are several groups of papers in this special issue. The first group is concerned about how different ecological factors (particularly family environment) are related to children and adolescent well-being. In the paper by Qi, Peng, and Sun, the association between social factors and Chinese children’s weight from the perspective of welfare mix framework was examined. While family welfare factor was represented by family living arrangements, the state welfare factor was represented by average government educational expenditure per student, and the market welfare factor was represented by GDP per capita. Results showed that family living arrangements were related to children’s weight. Besides, school-age children from areas with higher GDP per capita and higher governmental education expenditure were more likely to experience overweight and obesity. Another study in this area was conducted by Qi, Liu, Hua, Deng, and Zhou, focusing on how family assets influence multiple dimensions of child well-being by using a national-wild dataset of Chinese adolescents. Their findings suggest that the impacts of diverse household asset types varied across different aspects of children’s well-being. The findings underscored the importance of family asset-building to improve child welfare. Finally, Zhou, Ma, Du, Zhou, and Qi analyzed the relationship between housing conditions and adolescent socio-emotional well-being by using a large nationally representative dataset. They found that housing conditions were negatively correlated with adolescent depression and positively correlated with self-esteem.

In the second group of papers, we have collected several papers focusing on the relationship between parenting and child and adolescent well-being. First, Du, Jian, Hua, and Qi investigated the relationship between parenting styles and Chinese adolescents’ self-regulation learning. This study showed that self-esteem mediated the relationship between positive parenting styles and adolescents’ self-regulation learning. Second, in the longitudinal study conducted by Pan, Zhang, Liu, Li, and Liu, the relationship between parents’ attachment styles and Chinese adolescents’ regulatory emotional self-efficacy was examined. The findings showed that parental attachment anxiety influenced children’s attachment to parents, and the latter then affected children’s regulatory emotional self-efficacy. Third, in the paper by Zhang, Pan, Chen, Liu, Wang and Jean, the association between parent-child relationship and Chinese adolescents’ depressive symptoms was examined by using data from the China Education Panel Survey. Results indicated that while both good father-child and mother-child relationships reduced the risk of female adolescents’ depressive symptoms, only the father-adolescent relationship negatively predicted depression in male adolescents. Finally, Zhang, Cai, Peng, and Emery investigated the change of childcare arrangements in China in the past 20 years and its relationship with child health well-being. They found that during the past decades, the proportion of children with parents as primary caregiver decreased, and the proportion of children receiving grandparents’ daily care increased. Besides, children receiving care from formal childcare outside household had a higher height and lower BMI scores.

The third category of papers is on gender and ethnicity related to children and adolescent well-being. First, Wan and Gong examined the effect of psychological trauma on depression of children from different ethnic groups. They found that while child maltreatment was related to depression, the correlation of maltreatment and child depression was most salient among Han children and was least significant among Zhuang children. Second, Geng and He focused on gender differences in Chinese children’s psychological well-being. Results showed that girls showed higher psychological well-being than did boys. Besides, regardless of gender, while protective factors from family and school contributed to children’s psychological well-being, academic pressure and family conflict were related to poor psychological well-being. Finally, in Huang and Gong’s paper, researchers examined gender differences of the determinants of educational expectations among left-behind children. They found that left-behind boys had higher educational expectations than left-behind girls only during primary school. Besides, after entering middle school, the educational expectations of left-behind girls transcended the expectation of left-behind boys.

The last group of papers is concerned the left-behind children and migrant children. First, Fu and Chen conducted a study on the relationship between left-behind experience and children’s mental health by collecting data from 1,907 adolescents in western China. Results showed that children with both parents who had migrated had a higher score of depression than did children who had no left-behind experience, and paternal migration was also associated with left-behind children’s poor mental health. Second, Jiang, Duan, and Wang investigated how parental labor migration and its duration influenced children’s mental health using a national-wide dataset. They found that the mental health of children whose both parents had migrated was significantly poorer when compared with children from intact rural families. Besides, the migration duration also influenced children’s mental health. Third, in a meta-analysis conducted by Hu, Hu and Zhu, the association between Chinese migrant and left-behind children’s perceived discrimination and their mental health was examined. Results showed that perceived discrimination was negatively related to children’s mental health. Fourth, Liu, Deng, and Katz examined intergenerational transmission of educational outcomes across three generations of migrant children. Their findings suggested that grandparental socioeconomic status was positively associated with parental academic performance and education level, which was further positively related to migrant children’s academic achievements. Fifth, Cui and Xie examined how bullying victimization influenced Chinese migrant children’s mental health. Results showed that bullying victimization reduced children’s intrapersonal and interpersonal resilience, and the latter was consequently detrimental to children’s mental health. Finally, with the aim to promote left-behind children’s well-being, Xie, Chen, Cheung and Huang examined whether using intelligent robots could improve the health-related well-being of left-behind children. They found that children who used the robots showed a significantly higher level of health-related well-being, compared with children who did not use the robots.

We wish to take this opportunity to give our sincere thanks to the reviewers who provided valuable comments and constructive suggestions in reviewing the papers of this special issue guest edited by Prof. Huamin Peng and Dr. Zheng Zhou. Their contribution and support are indispensable as far as the quality assurance process of this special issue is concerned. The reviewers are: Prof. Julian Chun-Chung Chow from University of California, Berkeley; Prof. Joyce Ma from The Chinese University of Hong Kong; Prof. Xiaohe Xu from The University of Texas at San Antonio; Prof. Chien-Chung Huang from Rutgers University; Prof. Guo-sheng Deng from Tsinghua University; Prof. Bo Hu, from The London School of Economics and Political Science; Prof. Sang-Rok Lee from Jeonbuk National University; Prof. Ho-Geun Lee from Jeonbuk National University; Prof. Bingqin Li from University of New South Wales; Prof. Zai Liang from the State University of New York at Albany; Prof. Shouchui Zeng from East China University of Science and Technology; Dr. Heng Choon (Oliver) Chan from The City University of Hong Kong; Dr. Zvjezdana Prismic-Larsen from Washington University in St. Louis; Prof. Siu-ming To from The Chinese University of Hong Kong; Dr. Von Nebbitt from Washington University in St. Louis; Dr. Xiaoqin Zhu from The Hong Kong Polytechnic University; Dr. Diya Dou from The Hong Kong Polytechnic University; Dr. Zhiming Cheng from University of New South Wales; Dr. Wei Guo from Nanjing University; Dr. Yongqiang Zheng from George Fox University; Dr. Qian Zhang from Southwest University; Dr. Yunong Huang from Flinders University; Dr. Hao Lei from East China Normal University; Dr. Haining Wang from Sun Yat-Sen University; Dr. Jie Lei from Sun Yat-Sen University. We are much indebted to their critical and constructive reviews.
